# A streamlined strain engineering workflow with genome-wide screening detects enhanced protein secretion in *Komagataella phaffii*

**DOI:** 10.1038/s42003-022-03475-w

**Published:** 2022-06-08

**Authors:** Yoichiro Ito, Misa Ishigami, Goro Terai, Yasuyuki Nakamura, Noriko Hashiba, Teruyuki Nishi, Hikaru Nakazawa, Tomohisa Hasunuma, Kiyoshi Asai, Mitsuo Umetsu, Jun Ishii, Akihiko Kondo

**Affiliations:** 1 Engineering Biology Research Center, Kobe University, Kobe, Japan; 2 Graduate School of Science, Technology and Innovation, Kobe University, Kobe, Japan; 3 Technology Research Association of Highly Efficient Gene Design (TRAHED), Kobe, Japan; 4 Department of Computational Biology and Medical Sciences, Graduate School of Frontier Sciences, University of Tokyo, Chiba, Japan; 5 Bio-Pharma Research Laboratories, Kaneka Corporation, Takasago, Japan; 6 Department of Biomolecular Engineering, Graduate School of Engineering, Tohoku University, Sendai, Japan; 7 Department of Chemical Science and Engineering, Graduate School of Engineering, Kobe University, Kobe, Japan

**Keywords:** Applied microbiology, Industrial microbiology

## Abstract

Expression of secreted recombinant proteins burdens the protein secretion machinery, limiting production. Here, we describe an approach to improving protein production by the non-conventional yeast *Komagataella phaffii* comprised of genome-wide screening for effective gene disruptions, combining them in a single strain, and recovering growth reduction by adaptive evolution. For the screen, we designed a multiwell-formatted, streamlined workflow to high-throughput assay of secretion of a single-chain small antibody, which is cumbersome to detect but serves as a good model of proteins that are difficult to secrete. Using the consolidated screening system, we evaluated >19,000 mutant strains from a mutant library prepared by a modified random gene-disruption method, and identified six factors for which disruption led to increased antibody production. We then combined the disruptions, up to quadruple gene knockouts, which appeared to contribute independently, in a single strain and observed an additive effect. Target protein and promoter were basically interchangeable for the effects of knockout genes screened. We finally used adaptive evolution to recover reduced cell growth by multiple gene knockouts and examine the possibility for further enhancing protein secretion. Our successful, three-part approach holds promise as a method for improving protein production by non-conventional microorganisms.

## Abstract (web summary)

A high-throughput workflow involving a streamlined genome-wide screen improves protein production in the non-conventional yeast, *Komagataella phaffii*, following adaptive laboratory evolution.

## Introduction

The methylotrophic yeast *Komagataella phaffii*, commonly known as *Pichia pastoris*, is widely used as a producer of recombinant heterologous proteins of medical or industrial interest^1–5^. Contrary to many successes of this yeast, the severe burden that expression puts on the secretion pathway often leads to low productivity of target proteins^1,4,6^. Although a number of gene modifications have been suggested to directly or indirectly help (or impair) recombinant protein secretion in the eukaryotic model organism budding yeast *Saccharomyces cerevisiae*^7,8^, we do not yet understand the entire intracellular processes of protein secretion. Despite incomplete knowledge, overexpression or disruption of some genes (defined as gene-overexpression-type and disruption-type effective factors, respectively, in this study) has been reported to be beneficial for recombinant protein secretion^5,9–15^.

A genome-wide screen for mutant strains with enhanced protein secretion was reported^16^ by using a restriction enzyme-mediated integration (REMI)-based random mutation (genome-disruption) library^17–19^ in *K. phaffii*. The constitutive *GAPDH* promoter-driven β-galactosidase enzyme was chosen as a secretory protein model to utilize a growth selection pressure on lactose for ease of experiment. To improve protein secretion, one promising other strategy would be to combine multiple genetic modifications^12^. However, the studies typically combine the effective factors of well-known function in *K. phaffii* (e.g., overexpression of chaperons^20–22^ and secretion process-associated proteins^23,24^, knockout of protease^13^ and vacuolar protein sorting components^14,15^).

Adaptive laboratory evolution (ALE) is an another approach for improving microbial phenotypes in many organisms^25,26^. Several studies have reported that ALE can be used to improve cell growth reduced, and can result in an increase in the production of fermentative compounds^27–29^. However, successful applications of ALE to improving recombinant protein secretion have been rarely reported in *K. phaffii*^30^.

**Fig. 1:**
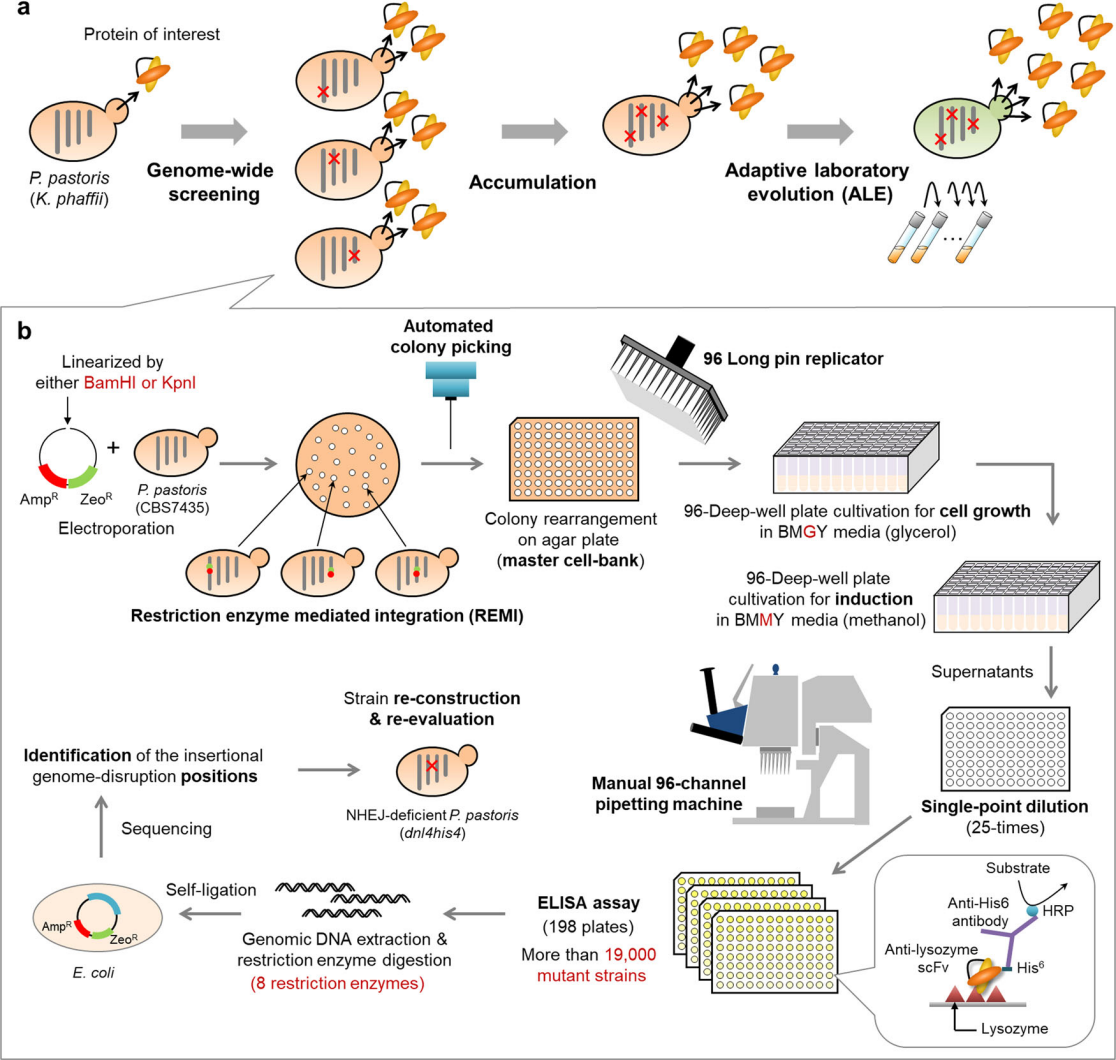
Strain development strategy for enhanced recombinant secretory protein production by *K. phaffii*. **a** Outline of our strain development scheme. Genome-wide high-throughput screening (HTS) for gene-disruption-type effective factors, and combining perturbations, can improve secretion of a target protein (e.g. small antibody), and adaptive laboratory evolution (ALE) can be used to improve cell growth and further increase protein production. A random genome-disruption library was constructed using restriction enzyme-mediated integration (REMI) and effective factors that increase the secretion titer of an anti-lysozyme scFv antibody (a model of difficult-to-secrete proteins) were identified. Red cross(es), genes whose disruption has beneficial effects on protein secretion. The factors can be combined to further improve protein secretion. The strains can then be subjected to ALE for further improvement. **b** Schematic of the high-throughput, genome-wide screening workflow. Linearized plasmids carrying resistance genes were electroporated into wild-type *K. phaffii* to generate a random genome-disruption library. These insertional genome-disrupted mutants were then arrayed to 96-well format on square agarose plates using an automated colony picker. The 96-well format transformants were then inoculated into glycerol media (BMGY) in 96-deep-well plates for cultivation, followed by inoculation into an induction medium (BMMY). The small antibody titers of individual genome-disruption strains (>19,000 strains) were determined from cleared culture supernatants by ELISA. Genome-disruption sites of positives were identified as follows: genomic DNA from positives was extracted, digested with 8 restriction enzymes, then self-ligated and transformed into *E. coli*. The sites of insertion were then determined by Sanger sequencing of plasmids extracted from *E. coli*. Gene-disruption strains independently generated via targeted homologous recombination were then evaluated to confirm correct identification of the gene-disruption-type effective factors.

In this work, we demonstrated that a combining genome-wide screening of gene-disruption-type effective factors, their accumulation in one strain and an ALE for recovery of the reduced cell growth of gene-knockout strains is an effective strategy for improving the secretion of heterologous proteins by the non-conventional yeast *K. phaffii* (Fig. 1a).

## Results

### A high-throughput screening system for enhanced small antibody secretion

As a first step, we set up a high-throughput screening (HTS) system, summarized in Fig. 1b, to easily evaluate the secretion of a small antibody by a large number of mutant strains. For this massive operation, all screening experiments were conducted in 96-deep-well plates and in a high-throughput manner. To link genotype (i.e. disruption of a particular gene) to phenotype (titer performance) for each REMI strain screened, we initially made master plates in which the mutated transformants were automatically re-arrayed in 96-well format using an automated colony picker. In addition, for high-throughput cultivation, 96 mutant strains at a time were inoculated into culture media in wells of 96-deep-well plates from the master plates using a 96-well format long pin replicator. With an aim to screen the mutant strains with high titers for the small antibody secretion, after pre-culture of the mutant strains with glycerol-containing media (BMGY) for full growth of each strain, an aliquot of each culture was transferred into methanol-containing media (BMMY) in each 96-deep-well plate to induce secretion of the small antibody. After cultivation, clarified supernatants from the 96-deep-well plates were collected to determine the antibody titer using a simplified enzyme-linked immunosorbent assay (ELISA) with a single-point dilution factor (25-fold dilution of the supernatants) using a 96-well liquid handling instrument and a simple microplate washer for treatment of multiple 96-well plates.

### Screening the random genome-disruption library

To identify gene-disruption-type effective factors that enhance secretory production of the target protein, we constructed a random genome-disruption library using a REMI technique^17–19^ with some modifications that can randomly integrate a linearized plasmid into genomic DNA via the innate non-homologous end joining (NHEJ) mechanism in *K. phaffii*. The *K. phaffii* wild-type strain CBS7435 was used as a host strain to elicit NHEJ-based random integration. As a model protein difficult to secrete, the scFv (V_H_-linker-V_L_) antibody for anti-lysozyme^31^ was chosen, and fused with the MFα pre-pro secretion leader peptide at the N-terminus and the His_6_-tag at the C-terminus of the scFv. The scFv gene was expressed under the control of a methanol-inducible *AOX1* promoter.

In a typical REMI approach, only a single restriction enzyme (e.g., *Bam*HI) is used for linearization of the integration plasmid. To construct a REMI library with a larger diversity, we separately used two restriction enzymes (*Bam*HI and *Kpn*I) to linearize the plasmid pREMI-ZA. The linearized plasmids were purified and then separately electroporated into a *K. phaffii* wild-type strain, resulting in randomly insertional genome-disrupted yeast libraries with a large diversity (total diversity: >10^5^, estimated from the number of transformants). To confirm that plasmid integration into *K. phaffii* in this library occurred at random, we performed a Southern blot analysis of 22 strains that were picked randomly from the two REMI-based libraries (linearized by either *Bam*HI or *Kpn*I). As shown in Supplementary Fig. 1, all of the bands from the *Bam*HI and *Kpn*I-linearized REMI strains had different mobilities, confirming random integration of the linearized REMI plasmid.

**Fig. 2:**
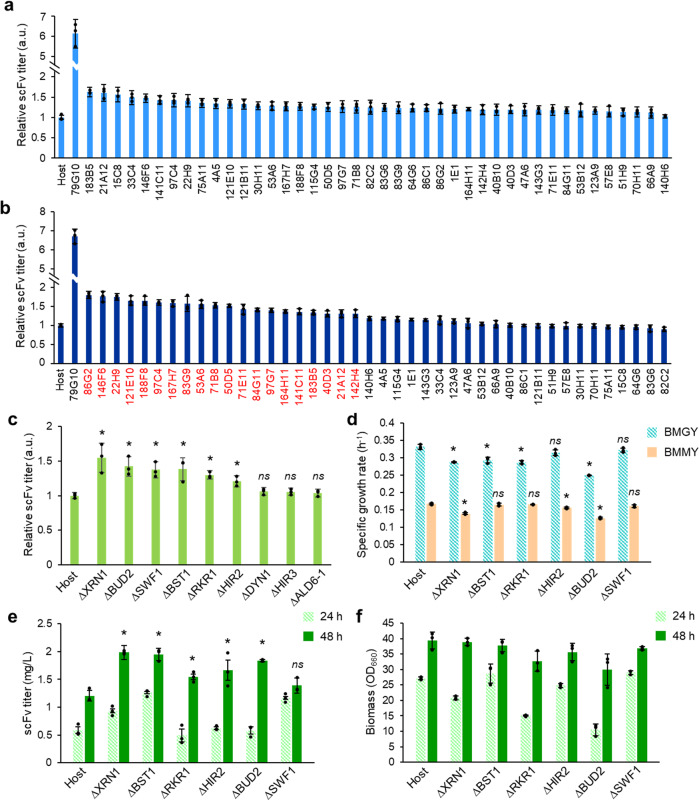
The scFv antibody titers from screening-positive clones. Relative titers of REMI-based screening positives cultured in 96-deep-well plates (**a**) or test tubes (**b**). A total of 43 positive strains were chosen from the REMI-based screen (**a**) and for re-evaluation (**b**) on the wild-type *K. phaffii* strain. A single highly positive and many weakly positive clones were obtained. The top 20 clones with higher protein secretion performance in test tube cultivation (1.3-times higher than the host strain) were labeled in red (except for 79G10, in which a mutation in the MFα secretion signal peptide contributed to the scFv secretion performance) (**b**). After identifying the plasmid integration locus for the 20 strains, nine unique gene disruptions were identified. The titer values of screening positives were normalized to that of the host strain. Error bars represent standard deviations from three technical replicates (**c**). Relative titers of test tube cultivations for *dnl4his4*–based re-construction strains at 60 h after methanol induction. Among the nine reconstructed strains, six gene-disrupted strains showed higher titers than the host strain with significant difference, indicating that six gene-disruption-type effective factors were identified. **d** Growth rate analysis of the six gene-disrupted strains and the host strain on BMGY (emerald, diagonal stripes) and BMMY (orange, filled bars) are indicated. **e**, **f** The re-constructed gene-disruption and the host strains were cultivated in the shake flasks. The titer (**e**) and final OD_660_ values (**f**) of each strain at 24 h (light green, diagonal stripes) and 48 h (dark green, filled bars) sampling points after methanol induction are indicated. Error bars represent standard deviations from three biological replicates. Asterisks and “*ns*” indicate the significance level (*p* < 0.05 by *t*-test) and non-significance level (*p* > 0.05 by *t*-test) between the host and effective factor-disruption strains, respectively.

Using our HTS system (Fig. 1b), we screened for yeast strains in which gene disruption resulted in higher secretion ability. Altogether, we screened more than 19,000 mutant strains (198 96-deep-well plates). Among these, we identified a single strain (79G10) with an antibody titer about 6-times higher than that of the host strain and 42 strains with 1.1 to 1.6-times higher scFv antibody secretion (Fig. 2a). For the highest-secretion strain, the scFv titer was reanalyzed following cultivation in a test tube cultivation and again, the results indicated that this strain secretes more than 6-times higher levels of scFv antibody than the host strain (Fig. 2b). However, the effect was not reproduced when we reconstructed a gene-disruption strain by REMI plasmid insertion into the genomic locus disrupted in strain 79G10 (Supplementary Fig. 2a). Thus, we performed whole-genome sequencing and analysis of the strain, and identified a V50A mutation on the MFα secretion signal of the scFv antibody. A re-constructed *K. phaffii* strain carrying the V50A mutation on MFα signal was associated with increased secretion of the small antibody as compared with the wild-type MFα signal strain (Supplementary Fig. 2b, c). A more detailed characterization of the mutation will be presented in another report^32^.

We also examined each of the 42 strains associated with slightly higher secretion (Fig. 2a). These were assayed for scFv titer following test tube cultivation and we observed 0.9 to 1.8-times higher secretion in each of these strains as compared with the host strain (Fig. 2b). Although there was a slight difference in the scFv titers from strains grown in 96-deep-well-plates versus in test tubes, we successfully confirmed that 20 strains had more than 1.3-times higher secretion ability as compared to the host strain in test tube cultivation (Fig. 2b). Thus, we defined these strains as positives from the screen and subjected them to a detailed analysis. This also demonstrated that our HTS approach worked reliably for the isolation of mutant strains with small but significant differences in scFv secretion ability from a massive strain library pool.

### Identification of gene-disruption-type effective factors

We next sought to identify what genes were disrupted in screen-positive strains and validate that disruption of the genes is associated with the improved protein production phenotype (Fig. 1b). To do this, we first determined the insertion sites of pREMI-ZA in the genomes of the screening-positive strains, then generated independent disruptions of these genes in the scFv secreting strain using targeted homologous recombination, and finally, compared the titers of secreted scFv in the insertional gene-disruption strain with that in the host strain. When we observed higher scFv secretion in the generated deletion strain as compared with the host, we concluded that the gene could correctly be defined as a gene-disruption-type effective factor.

An advantage of using a REMI-based method is the relative ease by which genome insertion sites can be identified^19^. Following this strategy (see Methods), we determined the pREMI-ZA integration sites for each of the 20 screening-positive clones. One restriction enzyme is typically used for genome cutting before self-ligation to obtain a plasmid comprised of genome-integrated REMI fragment and flanking chromosomal region^19^. In this experiment, however, we used eight restriction enzymes (all absent from pREMI-ZA) to generate short fragments of yeast genomic DNA. This allowed us to easily and efficiently isolate the plasmid. From sequence analysis of the genomic fragments flanking the pREMI-ZA sequences of the self-ligated plasmids, we were able to identify the genome insertion locus for 18 of 20 positives, and these comprised 13 unique gene disruptions (Table 1).

**Table 1:** Properties of higher scFv secreting strains obtained from the genome-wide screen.

Clone name	Disrupted gene	Locus tag	Protein ID	Gene product	Strain name**
50D5	XRN1	CHR3-0780	CCA39734	5’−3’ Exoribonuclease 1	RD-1
188F8	BST1	CHR2-0580	CCA38267	Glycosyl phosphatidyl inositol deacylase	RD-2
164H11	RKR1	CHR1-1469	CCA37582	E3 ubiquitin-protein ligase	RD-3
53A6, 141C11	HIR2	CHR2-0260	CCA37956	HIR (histone regulatory) complex subunit	RD-4
40D3	BUD2	CHR3-0544	CCA39503	GTPase activating factor	RD-5
146F6	SWF1	CHR2-1252	*	Palmitoyltransferase	RD-6
142H2, 183B5	ALD6-1	CHR4-0972	CCA41122	Magnesium-activated aldehyde dehydrogenase, cytosolic	RD-7
121E10	HIR3	CHR2-0471	CCA38159	HIR complex subunit	RD-8
71E11, 83G9, 167H7	DYN1	CHR2-0532	*	Dynein heavy chain	RD-9
22H9	NUM1	CHR1-0804	CCA36944	Mitochondria-ER cortex anchor component	–
86G2, 97C4	HypoP-1	CHR1-0785	CCA36925	Hypothetical protein	–
97G7	HypoP-2	CHR1-1238	CCA37362	Hypothetical protein	–
71B8	SPT6	CHR4-0688	CCA40846	Nucleosome remodeling protein	–
84G11	Not determined				
21A12	Not determined				

*Not identified at NCBI (https://www.ncbi.nlm.nih.gov/).

** Re-constructed *Komagataella phaffii dnl4his4* strains carrying scFv expression cassettes with gene deletions. Nine deletion strains were obtained in this study.

To validate the 13 unique gene disruptions, we built engineered yeast strains with complete (null) gene disruptions of each potential effective factor by double-crossover homologous recombination using DNA fragments that contained an hygromycin resistance gene and flanking up- and downstream fragments (0.3 to 1.2 kb) of the target genes. To do this, we used a NHEJ-deficient *K. phaffii dnl4his4* strain^33^ (*DNL4*-disrupted strain; encoding DNA ligase IV), which results in more precise homologous recombination of the selective cassette into the target locus, and generated a *dnl4his4* strain with *AOX1* promoter-driven scFv secretory expression cassette. Through targeted gene disruption using the *dnl4his4* scFv secreting strain as the parental strain, 9 disruption strains were successfully generated.

We next evaluated secretion titers of the scFv from test tube cultivations of the nine successful gene deletion strains and the parental (host) *dnl4his4* strain. Prior to this experiment, we have confirmed that the scFv titer of *dnl4his4* strain was 1.3-times higher than that of CBS7435 wild-type strain (Supplementary Fig. 3). As a consequence, we chose the strain derived from *dnl4his4* for further investigations in this study. The scFv titers of six deletion strains were significantly higher than that of the host *dnl4his4* strain (1.2 to 1.6-times, *p* < 0.05, Fig. 2c). This indicated that we successfully identified six single gene-disruption-type effective factors, each of which can enhance scFv secretion. Detailed information about these factors is presented in Supplementary Data 1. With the exception of *XRN1*^34^, these factors had not been previously reported. In brief, Bst1p works as a negative regulator of coat protein complex II (COPII) vesicle formation^35^, such that the *BST1* disrupted strain would be expected to have increased the formation of COPII vesicles and improved transportation of target proteins between the endoplasmic reticulum (ER) and the Golgi apparatus. Rkr1p works as ‘Really Interesting New Gene (RING)’ domain of E3 ubiquitin ligase^36^. Thus, disruption of *RKR1* might inhibit ubiquitination of the target heterologous proteins, resulting in avoidance of ubiquitin-mediated protein degradation. The mechanism underlying the impact of the other three factors, Hir2p, Bud2p, and Swf1p, cannot be easily explained based on the information previously reported about these genes (Supplementary Data 1). Notably, these gene disruptions, however, appear to contribute to the protein secretory production by different mechanisms.

We next evaluated the growth rate of these six gene deletion strains on both glycerol (BMGY) and methanol media (BMMY), and scFv mRNA levels on methanol induction media (BMMY). Both the growth rates and the mRNA levels were almost equal or slightly lower as compared with those of the host strain (Fig. 2d, Supplementary Fig. 4). Thus, the higher performance of these deletion strains with regards to scFv secretory production cannot simply be attributed to enhanced cell growth or transcription.

### Combining gene-disruption-type effective factors in a single strain

Although we could not obtain extremely high secretion with a single gene disruption, we did identify several independent factors that reproducibly improved secretion titers. To further increase production, we combined these gene disruptions in a single strain. Five antibiotic resistance genes are available for *K. phaffii*. One of these, G418, is used in the scFv expression cassette. Thus, we can introduce into the *dnl4his4* strain four gene disruptions using a strategy based on homologous recombination of selection cassettes. To choose four factors among the six candidates, we first characterized the performance of the six gene-disruption strains in shake flask cultivation (Fig. 2e, f). We then chose four factors, *XRN1*, *BST1*, *RKR1*, and *HIR2*, as they had higher scFv secretion titers and comparatively higher biomass (OD_660_).

**Fig. 3:**
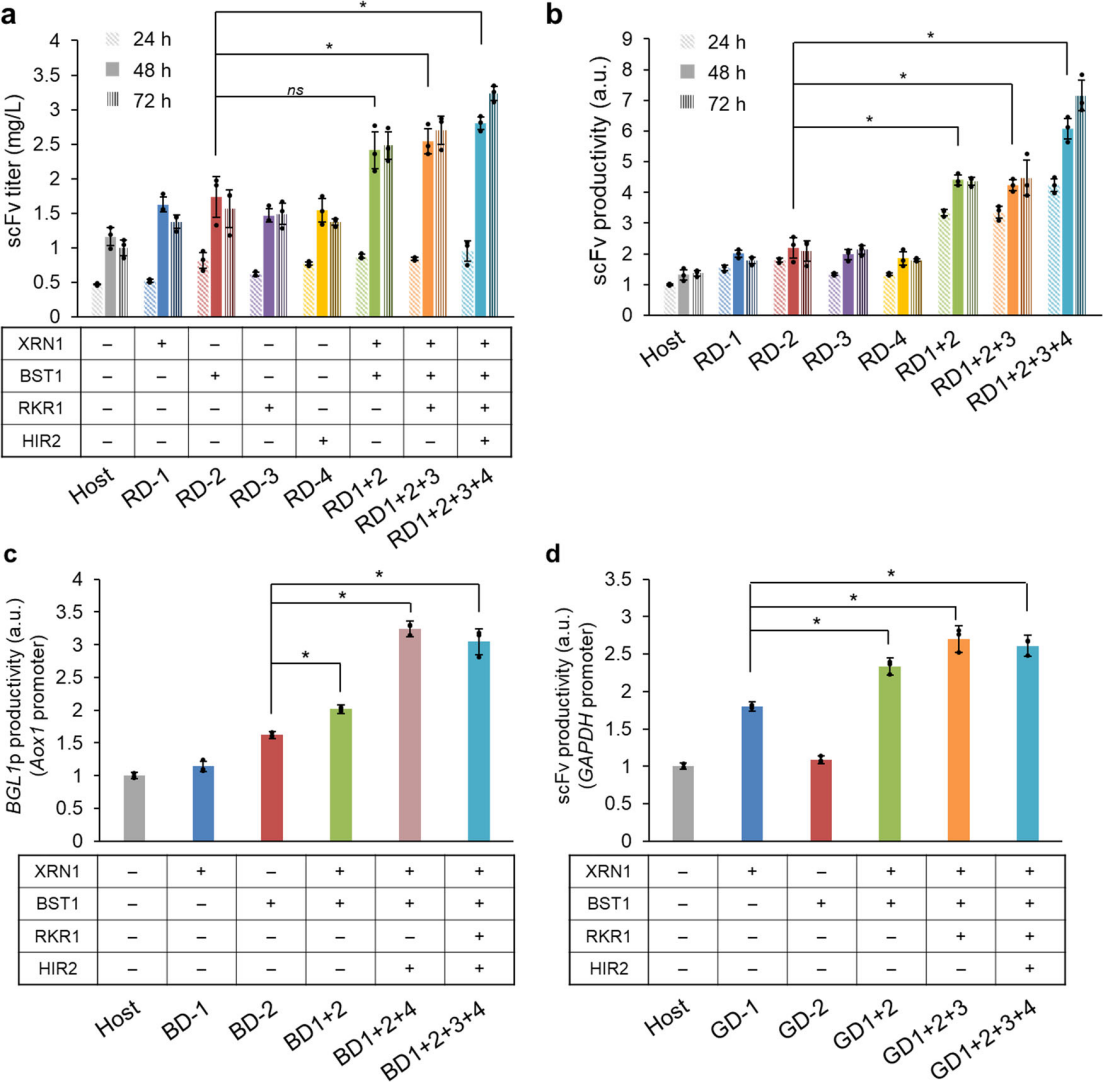
Combined perturbation of gene-disruption-type effective factors in a single strain. **a**, **b** The scFv titer (**a**) and the productivity (**b**) (scFv titer per OD_660_ value) in shake flask cultivation at 24 h (diagonal stripes), 48 h (filled bars) and 72 h sampling (vertical stripes) after methanol induction are indicated. For each timepoint, scFv productivity was normalized to that of the host strain at 24 h. The scFv productivity of the multi-deletion strains (RD1 + 2, RD1 + 2 + 3 and RD1 + 2 + 3 + 4) were significantly higher than that of the single effective factor-disruption and the host strains. Error bars represent standard deviations from three biological replicates. Asterisks and “*ns*” indicate the significance level (*p* < 0.05 by *t*-test) and non-significance level (*p* > 0.05 by *t*-test) between the RD-2 strain (the best secretion strain among single effective factor disruptions) and the accumulated strains at 48 h methanol induction, respectively. **c**, **d** Effects of swapping of the secreted protein and promoter in combined gene-disruption-type effective factor strains. Relative productivities (BGL1p activity or scFv titer per final OD_660_ value) of host and gene-disruption(s) strains in test tube cultivation for 60 h are indicated. (**c**) *AOX1* promoter-driven BGL1 and (**d**) *GAPDH* constitutive promoter-driven anti-lysozyme scFv constructs are indicated. Each productivity value was normalized to that of the host strain. Gray, blue, red, purple, yellow, yellow-green, orange and blue-green indicate the host, RD-1, RD-2, RD-3, RD-4, RD1 + 2, RD1 + 2 + 3 and RD1 + 2 + 3 + 4 strains, respectively. Error bars represent standard deviation from three biological replicates. Asterisks indicate that a significant difference was observed (*p* < 0.05 by *t*-test) in the BD-2 or GD-1 strain (the best secretion strains among single effective factor disruptions) and the multi-gene-disrupted strains.

To next ask if combining gene disruptions improved scFv secretion, three strains (RD1 + 2, RD1 + 2 + 3, RD1 + 2 + 3 + 4; RD, *AOX1* promoter-driven scFv secreting strains screened by random disruption) were constructed by sequential disruption of *XRN1*, *RKR1* and *HIR2* using different selective markers (nourseothricin, Zeocin and blasticidin resistance genes, respectively) into the *BST1* disrupted strain (RD-2 strain, hygromycin resistance gene). The scFv titers, the biomass and the productivities (scFv titer / OD_660_) of these strains were then compared with the host strain and each reconstructed single effective factor strain (RD-1, RD-2, RD-3, and RD-4; Table 1). The results obtained with these combined-deletion strains show that the scFv titers gradually but steadily increased (Fig. 3a), although biomass (OD_660_) decreased with the addition of each gene disruption (Supplementary Fig. 5). The strain with four gene disruptions (RD1 + 2 + 3 + 4) was the best producer and achieved approximately 3- and 5-times higher scFv titers and productivity, respectively, as compared with the host (Fig. 3a, b). In addition, both the scFv titers and the productivities for the three combination gene-disruption-type effective factor strains (RD1 + 2, RD1 + 2 + 3, RD1 + 2 + 3 + 4) were higher than the host strain or single-disrupted strains (*p* < 0.05; for simplicity, only the significance levels between RD-2 and accumulated strains are shown in Fig. 3a, b). Together, these data show that the strategy of combining gene disruptions was beneficial to the secretion of anti-lysozyme scFv expressed in *K. phaffii* under the control of the *AOX1* promoter.

### The effects of multi-gene disruption are independent of protein and promoter

We next asked if the effect we observe in the combined gene-disruption strain is specific to the *AOX1* promoter and/or this scFv, or if the effect is general. To do this, we exchanged the target protein and promoter. We chose *Aspergillus aculeatus* β-glucosidase (BGL1p)^37^ (under the control of the *AOX1* promoter) as an alternative secreted protein, and the constitutive *GAPDH* promoter as an alternative promoter (for scFv). Using homologous recombination with antibiotic cassettes as described above, the four effective factors were sequentially disrupted in *dnl4his4* strains containing the alternative expression cassettes (i.e. BGL1p secretion controlled by inducible *AOX1* promoter or scFv secretion controlled by a constitutive *GAPDH* promoter). Specifically, we constructed two single gene-disruption strains (*XRN1* or *BST1* was disrupted; BD-1 and BD-2 (for BGL1p; BD, *AOX1* promoter-driven BGL1p secreting strains with gene disruption), or GD-1 and GD-2 (for scFv; GD, *GAPDH* promoter-driven scFv secreting strains with gene disruption), respectively), and three multi-gene-disruption strains (*XRN1*, *BST1*, *HIR2* and *RKR1*, or *XRN1*, *BST1*, *RKR1* and *HIR2* were sequentially disrupted; BD1 + 2, BD1 + 2 + 4 and BD1 + 2 + 3 + 4 (for BGL1p), or GD1 + 2, GD1 + 2 + 3 and GD1 + 2 + 3 + 4 (for scFv), respectively). These strains were cultured in BMMY (methanol) or BMGY (glycerol) media in test tubes, then protein secretion was evaluated.

We define BGL1p productivity as the relative value of BGL1p activity (absorbance at 405 nm) divided by the final OD_660_ for each strain as compared to the host strain (*AOX1* promoter). Both BGL1p and scFv (*GAPDH* promoter) were higher in the multi-disruption strain than in the single-gene-disrupted or host strains (*p* < 0.05, Fig. 3c, d), although there were slight differences in scFv secretion under control of *AOX1* (Fig. 3b). The *XRN1* single gene disruption in the BGL1p secretion background (BD-1) and the *BST1* single gene disruption in the scFv secretion background with the *GAPDH* promoter (GD-2) showed lower enhancement of BGL1p (Fig. 3c) or scFv (Fig. 3d) productivity, respectively. In addition, the strains with three gene disruptions (BD1 + 2 + 4 and GD1 + 2 + 3) had almost the same productivities and titers as those with four, while the strains four gene disruptions had slightly higher biomass (final OD_660_) than strains with three (Fig. 3c, d and Supplementary Fig.6). The performance of the effective factors for protein secretion and the final OD_660_ seemed to be dependent on which protein was secreted and on the expression system (Supplementary Fig.6). Nevertheless, these data support the idea that combining perturbation of the gene-disruption-type effective factors in a single strain led to a moderate improvement and thus, might be effectively applied to other protein secretion and expression systems as well.

### Adaptive laboratory evolution (ALE) of the multiple gene-disruption strains

Finally, we asked if we could improve the expression system by increasing fitness of the multiple gene-disrupted strain. To do this, we used an adaptive laboratory evolution (ALE). A representative subset of five yeast strains with multiple gene disruptions (RD-1, RD1 + 2, RD1 + 2 + 3, RD1 + 2 + 3 + 4 and the host) was repeatedly grown in YPG (glycerol) media and inoculated into fresh glycerol media in test tubes, even though a methanol inducible construct was used for scFv secretion in this study. To the best of our knowledge, there exists only one report of a successful ALE experiment for improving protein production in *K. phaffii*^30^. In that report, methanol media was used for long-term carbon source adaptation in a wild-type strain^30^, whereas in this study, we used a glycerol medium for ALE of gene-knockout strains. To avoid contamination, YPG rich medium supplemented with appropriate antibiotics (“YPG + ”) was used as the glycerol media for the consecutive transfers experiment because the antibiotics cannot work in BMGY medium, which was used for culture in the protein production experiment. Approximately 0.2% (v/v) of each culture (host, RD-1 and RD1 + 2 strains) was serially transferred to fresh media daily, or every 1.5 days (for the RD1 + 2 + 3 and RD1 + 2 + 3 + 4 strains), until each culture had been transferred 55-times (approximately 500 generations). To characterize a single clone of evolved strains, four colonies were randomly isolated from the final 55-times transfer point of the continuous cultured (evolved) population and tested in both the long-term cultured YPG + media and the inductive methanol-containing BMMY media. The evolved populations, isolated evolved strains and non-continuous cultured (ancestral) strains for the four gene-disruption(s) and host strains were evaluated for scFv titer, biomass, and specific growth rate under both YPG + and BMMY conditions.

**Fig. 4:**
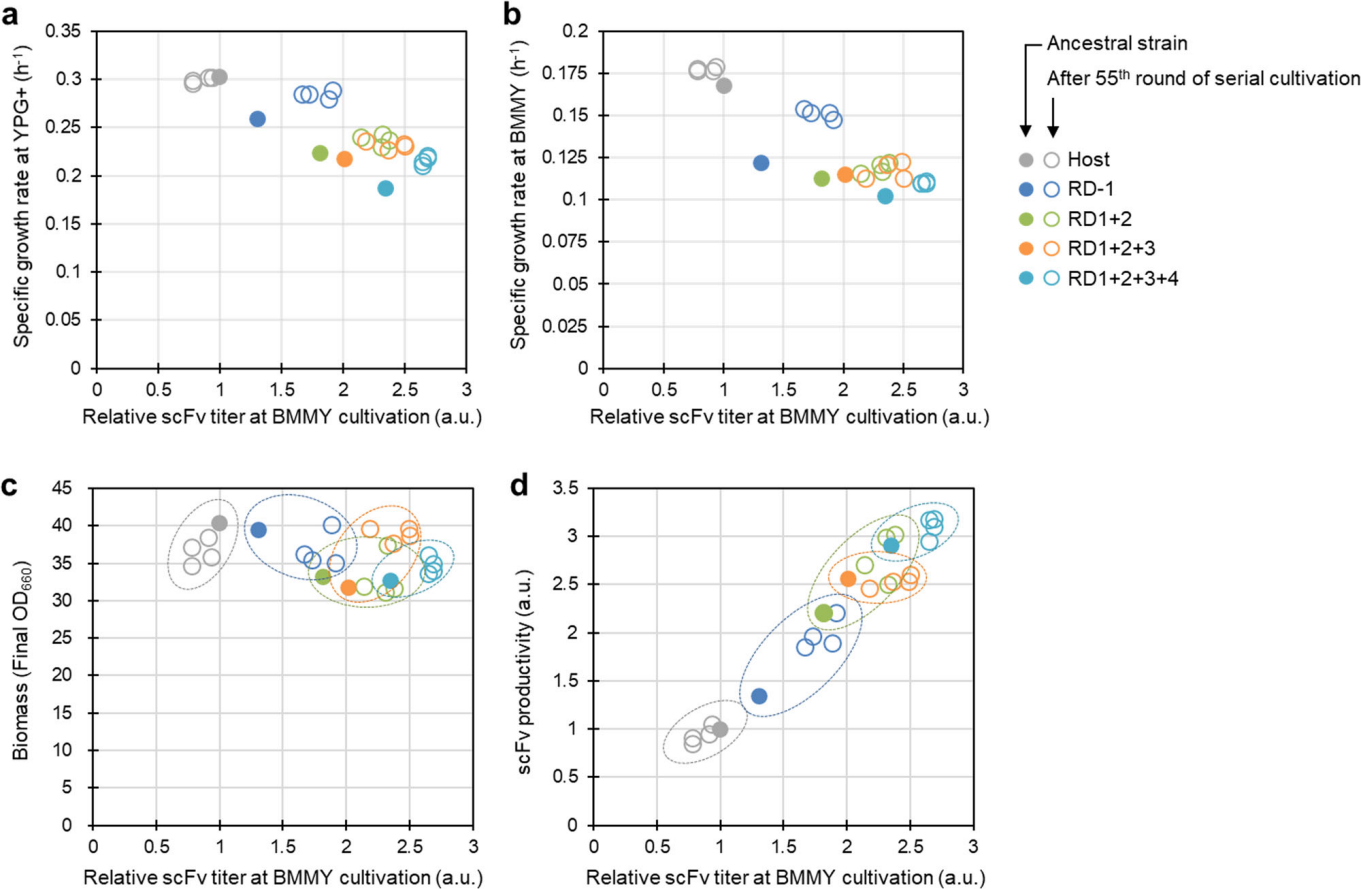
Properties of the ancestral and evolved cells in the host and multiple gene-disruption-type effective factor(s) strains. **a**, **b** Specific growth rate vs. relative scFv titer. Specific growth rates of the ancestral strains and the evolved strains (four isolated colonies) were evaluated in YPG media supplemented with proper antibiotic(s) (YPG+) media (**a**) and BMMY media (**b**) after the YPG + pre-cultivation. The relative scFv titers were evaluated by ELISA of supernatant of the test tube culture (pre-culture at BMGY and scFv induction at BMMY for 48 h). Adaptive laboratory evolution (ALE) was conducted by 55 rounds of serial cultivation in glycerol media (YPG+). Each of the (multiple) gene-disruption(s) and host strains was inoculated into YPG+. After cultivation at 30 °C for 24 h, a small aliquot of culture was inoculated to YPG + (initial OD_660_: 0.02) (**a**) and BMMY (initial OD_660_: 0.05) (**b**) followed by cultivation at 30 °C with a Bio-photorecorder. Relative scFv titer vs biomass (final OD_660_) (**c**) and relative productivity (d) of the ancestral and evolved strains (four isolated colonies) grown in BMMY media for 48 h are shown. Filled and open circles indicate the ancestral strain and four isolated strains after the 55^th^-serial cultivation, respectively. Dashed circles indicate directions of the ALE for each strain. Gray, blue, yellow-green, orange and blue-green indicate the host, RD-1, RD1 + 2, RD1 + 2 + 3 and RD1 + 2 + 3 + 4 strains, respectively. Each titer and productivity value was normalized to that of the ancestral host strain. Error bars of represent standard deviation from three technical replicates.

For the ancestral strains (without long-term cultivation), the specific growth rates decreased as the number of gene disruptions increased, both in YPG + and in BMMY (Fig. 4a, b and Supplementary Fig. 6a, b). Except for RD1 + 2, the evolved (55-times transferred) strains had higher specific growth rates than the corresponding ancestral strains in YPG + media. In contrast, the specific growth rate of the host strain did not change (Fig. 4a). In BMMY media, the host and gene-disrupted strains showed nearly the same but slightly higher specific growth rates as the ancestral strains after evolution, with the exception of the RD-1 strain, which exhibited an obviously higher specific growth rate in BMMY media after evolution (Fig. 4b). These results indicated that ALE had an incomplete but positive effect on the reduced cell growth in glycerol media of the multiple gene-disruption-type effective factors strains, resulting in recovery of cell growth even in methanol media, despite the fact that methanol media was not used in the adaptive long-term cultivation (Fig. 4a, b).

Next, the scFv titer, biomass and productivity of the ancestral and evolved four gene-disruption(s) and host strains in BMMY (methanol) media were evaluated at the point of the final 55^th^ transfer (Fig. 4c, d and Supplementary Fig. 7). Interestingly, the evolved gene-disrupted strains had on average 1.1 to 1.4-times higher scFv secretion abilities than the corresponding ancestral strains (*p* < 0.05), although the evolved host strains showed slightly lower scFv titers than the ancestral strain (Fig. 4 and Supplementary Fig. 7c). The biomass (final OD_660_) among the evolved strains grown in BMMY media varied and was slightly higher or lower than the ancestral strains except for the RD1 + 2 + 3 (Fig. 4c), while the specific growth rates of all strains in BMMY media slightly increased after the adaptive continuous cultivations (Supplementary Fig. 7b). The scFv productivities (scFv titer per biomass) of the evolved gene-disrupted strains (except for RD1 + 2 + 3 and the host) were higher than that of the ancestral strain (*p* < 0.05, Supplementary Fig. 7e). Since the biomass of the evolved RD1 + 2 + 3 strain was much higher than that of the ancestral strain (*p* < 0.05), its productivity was not improved, despite the increased titer of scFv (Fig. 4c, d). These data indicate that there were three routes to acquiring higher scFv titer by ALE: (i) recovery of biomass (RD1 + 2 + 3), (ii) acceleration of scFv productivity (RD-1 and RD1 + 2), and (iii) both (RD1 + 2 + 3 + 4). These improvements in cell growth and protein secretion might result from rewiring of metabolic or regulatory networks affected by the gene disruption(s)^27,28^, although further work needs to be done to understand the mechanisms underlying these improvements in fitness.

## Discussion

In this study, we successfully demonstrated as a proof of concept that a strategy comprised of genome-wide screening, combining gene-disruption-type effective factors identified in the screen, and ALE of the growth-reduced multiple gene-disruption strains improved secretory production of target recombinant proteins.

To identify gene-disruption-type effective factors that enhance scFv secretion, we modified the REMI-based random genome disruption technique and established a streamlined HTS system that allowed us to directly evaluate small antibody secretory production using a 96-well format workflow that included automated colony picking, collective inoculation and cultivation, and a simplified ELISA. Using this system, we successfully identified six gene-disruption-type effective factors (genes) for which knockout improves scFv secretion in *K. phaffii*, although some gene-disruption strains showed lower growth rate at the cultivation of BMGY and BMMY. In general, there have been reports that the effective factors are highly dependent on the target protein sequences^12,16,38^; however, we found that the multiple combination of them was applicable to the three constructs we tested (swapping secretory protein or promoter), probably owing that the effective factors with different aspects of the secretion process^3,10,24^ might be selected dispersedly in a genome-wide screen^39^. CRISPR-based genome editing^40,41^ and base editing technologies^42^ have great potential to make (multiple) gene disruption(s) more efficient, although these technologies are at present hard to use in *K. phaffii* due to a need for correct gRNA design with ribozymes against each target^43^.

Recently, excellent HTS assay methods in *S. cerevisiae* was also reported by using combinations of microfluidic droplet screening for random mutagenesis libraries by UV radiation^44^ or a random gene attenuation library by RNA interference^45^, and next-generation sequencing. Although the microfluidic screening allowed the higher library size at once (more than 10^5^), the target protein of these HTS was limited to the enzymes (e.g., α-amylase) which had their fluorogenic substrate to measure titers of the enzyme secreted in individual droplets by flowcytometry. On the other hands, our streamlined HTS system based on the automated colony picking and simplified ELISA is widely applicable to various proteins including enzymes and antibodies in a reliable and reproducible manner, while the throughput makes concession to the droplet systems.

Our ALE results indicate that the evolved strains with (multiple) gene-disruption(s) experienced a slight but steady recovery of cell growth rates not only in YPG + but also in BMMY (methanol) media, associated with a small enhancement in scFv secretory production, demonstrating the merits of applying an ALE strategy to improve production of recombinant proteins by the growth-reduced gene-knockout strains. Altogether, our three-part strategy of genome-wide screening, combining gene-disruption-type effective factors in a single strain, and ALE adaptation for improved cell growth in *K. phaffii* provides a success of this approach and shows promise for strain development aimed at improving recombinant protein secretory production in the non-conventional microbes with little known information available.

## Methods

### Strains and media condition

The wild-type *K. phaffii* strain CBS7435 (NRRL-Y11430) was used in the REMI screen. The *K. phaffii* strain *dnl4his4*^33^ was used for accurate gene disruptions in the effective factor validation and multiple-disruption processes. *E. coli* strain DH5α was used for recombinant DNA manipulation.

*K. phaffii* strains were grown in YPD or YPG media [10 g/L yeast extract (Nacalai Tesque, Kyoto, Japan), 20 g/L Bacto peptone (BD Biosciences, San Jose, CA, USA) and 20 g/L glucose (for YPD) or 20 g/L glycerol (for YPG)], or in BMGY or BMMY media [10 g/L yeast extract, 20 g/L hipolypeptone (Nihon Pharmaceutical, Tokyo, Japan), 13.4 g/L yeast nitrogen base without amino acids (BD Biosciences), 0.4 mg/L biotin (Nacalai Tesque), 100 mM potassium phosphate buffer (final, pH 6.0) and 20 g/L glycerol (for BMGY) or 20 g/L methanol (for BMMY)]. *E. coli* strains were grown in LB media with 5 g/L yeast extract, 10 g/L tryptone (Nacalai Tesque) and 5 g/L NaCl, supplemented with ampicillin (100 μg/mL). YPD agar plate contained 20 g/L agar in YPD media with antibiotics (500 μg/mL G418 (Wako Pure Chemical Industries, Osaka, Japan), 100 μg/mL Zeocin (InvivoGen, San Diego, CA, USA), 100 μg/mL hygromycin (Wako Pure Chemical Industries), 50 μg/mL nourseothricin (Werner BioAgents, Jena, Germany), and/or 100 μg/mL blasticidin (Wako Pure Chemical Industries)). Square YPD plates contained 100 μg/mL Zeocin.

### Construction of plasmids and *K. phaffii* strains

The plasmids and yeast strains used in this study are listed in Supplementary Data 2 and 3, respectively. The primers and synthetic DNAs used in this study are listed in Supplementary Data 4 and 5, respectively. Primers were purchased from Eurofins Genomics K.K. (Tokyo, Japan) and synthetic DNA fragments were synthesized as GeneArt Strings DNA Fragments (Thermo Fisher Scientific, Waltham, MA, USA) with codon usage optimized for *K. phaffii*. Standard recombinant DNA manipulation was performed as described by Sambrook et al.^46^.

The gene encoding the single-chain variable fragment (scFv, VH-linker-VL) antibody for anti-lysozyme^31^ with a His_6_ tag at the C-terminus of the scFv and the MFα pre-pro-leader from *Saccharomyces cerevisiae* as a secretion signal sequence was synthesized and inserted in place of the *EGFP* gene into the plasmid pPGP_EGFP^33^, to prepare the plasmid pPGP_scFv. The DNA fragment of *AOX1* promoter was amplified by PCR using the genome of the *K. phaffii* wild-type strain CBS7435 as a template. The plasmid pPAP_scFv was constructed from pPGP_scFv by replacing the *GAPDH* promoter fragment with the *AOX1* promoter via ligation at the *Bam*HI/*Spe*I sites. To prepare reconstruct the V50A mutation on the MFα signal sequence in pPAP_scFv, generating pPAP_scFv_V50A, the DNA fragment of the MFα signal sequence was amplified by PCR using the genome of the selected 79G10 strain as a template, and then replaced the MFα signal sequence of pPGP_scFv with the PCR amplified MFα signal sequence, including the V50A mutation, using Infusion technology (Takara Bio, Shiga, Japan). A DNA fragment containing a secretion signal sequence derived from *Rhizopus oryzae* glucoamylase (SSRG) and a BGL1p-encoding ORF^47^ was amplified by PCR. The plasmid pPAP_BGL1 was constructed by exchanging the scFv gene of pPAP_scFv for the PCR-amplified SSRG-*BGL1* ORF using Infusion technology (Takara Bio). The REMI plasmid pREMI-ZA was constructed based on pUC19. A DNA fragment of the zeocin resistant gene was inserted on the *Eco*RI site of pUC19 using Infusion technology following disruption with *Eco*RI. All plasmids constructed in this study were confirmed by Sanger sequencing.

The host yeast strains for scFv or BGL1p secretion were generated by a plasmid integration of the *Eco*RV linearized pPAP_scFv (for scFv expression by the *AOX1* promoter), pPGP_scFv (for scFv expression driven by the *GAPDH* promoter) or pPAP_BGL1 (for BGL1p secretion by the *AOX1* promoter) into the 3’-UTR region of CCA38473 gene of the wild-type *K. phaffii* strain CBS7435 (for preparing REMI library) or the *dnl4his4* strain (host for targeted gene-disruption) using the lithium acetate transformation method described below, for preparation of the strains CBS7435_scFv, *dnl4his4*_Pgapdh-scFv and *dnl4his4*_BGL1, respectively. Single copy integration of each linearized plasmid into the correct locus was verified by qPCR and colony PCR using appropriate primers (Supplementary Data 4).

### Lithium acetate transformation

Lithium acetate transformation was conducted using a previously reported method^33,48^. Briefly, yeast cells were grown in YPD medium overnight, subsequently diluted 10-fold with fresh YPD medium, and then cultured for an additional 4 h. After the 4-h culture, the cells were pelleted, washed with deionized water, and resuspended in a transformation mix comprising 120 μL of 60% PEG3350, 5 μL of 4 M lithium acetate, 10 μL of 1 M DTT, and 10 μL of 10 mg/mL carrier DNA (Yeastmaker carrier DNA; Takara Bio) and containing 2 μg of the linearized plasmid DNA. This mixture was incubated at 42 °C for 30 min, followed by the addition of 1 mL of YPD medium and further incubation at 30 °C for 2 h. The resultant transformants were spread on YPD agar plates that contained the proper antibiotics.

### Construction and evaluation of a REMI library

A random genome-disruption library (REMI library) was prepared as follows. Construction of REMI library basically followed Mukaiyama et al.^19^ with slight modifications. The plasmid pREMI_ZA was separately linearized with *Bam*HI or *Kpn*I, and DNA purified using the Wizard SV Gel and PCR Clean-Up System (Promega, Madison, WI, USA), followed by electroporation into the wild-type host strain carrying the scFv gene driven by *AOX1* promoter (CBS7435_scFv) and spread out on YPD plate supplemented with Zeocin (100 μg/mL). Transformants of the REMI library were visible after incubation at 30 °C for 2 days.

Randomness of the REMI integration into the *K. phaffii* genome was confirmed by Southern blot analysis. In detail, genomic DNA from randomly picked yeast clones in the REMI library were extracted using a genomic DNA extraction kit (Dr. Gentle, Takara Bio), and then digested with *Eco*RI. Each sample was then subjected to agarose gel electrophoresis (1% agarose gel) followed by Southern blot analysis using the DIG-High Prime DNA Labeling and Detection Starter Kit II (Roche Life Science, Penzberg, Germany) following the manufacturer’s instructions. A PCR amplified DNA fragment (300 bp) of the Zeocin resistance gene was used as a probe.

### High-throughput screen of a random genome-disrupted library

A high-throughput screen of random genome-disrupted yeast strains in 96-deep-well plates was conducted as depicted in Fig. 1b. Transformants of a REMI library were aligned in 96-well format on YPD square plates supplemented with Zeocin (100 μg/mL) using an automated colony picker (PM-2 multi, Microtec Co., Ltd., Chiba, Japan). After incubation at 30 °C for 2 days, the 96-well format colonies were inoculated simultaneously into 500 μL of BMGY (glycerol) media in each well of a 96-deep-well plate (#3960, Corning, Corning, NY, USA) using a 96-long pin replicator (96 Copy Plate Replicator, TK-CP96, Tokken Inc., Chiba, Japan). Following pre-cultivation on a plate shaker (M•BR-420FL, TAITEC Corp., Saitama, Japan) at 30 °C for 24 h with orbital shaking at 1500 rpm, 50 μL of the cultures was inoculated into 500 μL of BMMY (methanol) media in the 96-deep-well plate, and cultured at 30 °C for 48 h with shaking at 1500 rpm for methanol induction of the scFv secretion. The scFv titer of each yeast strain was measured by a simplified ELISA using a 96-channel pipette (Liquidator, Mettler-Toledo, Columbus, OH) and a plate washer (Wellwash, Thermo Fisher Scientific) for easy manipulation of a large number of plates.

### ELISA quantification of scFv secretion titer

A simplified sandwich ELISA was used to determine the titer of secreted scFv antibody from culture supernatants. Immunosorbent plates (96-well format, #3369, Corning) were coated with 50 μL of 1 μg/mL lysozyme from egg white (Wako Pure Chemical Industries) in phosphate-buffered saline (PBS), left at 4 °C overnight, and then blocked at 4 °C overnight with 5× diluted ImmunoBlock (DS Pharma Biomedical, Osaka, Japan). The ELISA plates were subsequently washed three times with PBT wash buffer (PBS with 0.1% Tween20). Fifty microliters of culture supernatants diluted 25× in dilution buffer (PBST with 2% ImmunoBlock) was added to one lysozyme-coated well of the 96-well plate. The supernatants were obtained by centrifugation of each yeast culture at 3500 × *g* for 5 min. The standard used for quantification was purified anti-lysozyme scFv. Following incubation for 1 h at room temperature, the samples were washed four times with wash buffer, and the captured scFv was subjected to another incubation for 1 h at room temperature with 50 μL of 100,000× diluted mouse anti-His antibody conjugated with horseradish peroxidase (Abcam, Cambridge, UK). After five washes with wash buffer, the samples were stained with SureBlue TMB 1-component Microwell Peroxidase Substrate (SeraCare Life Sciences, Milford, MA, USA). Fifty microliters of 1 N HCl was added after 3 min to stop the color reaction, and then adsorption at 450 nm was measured using an Envision microplate reader (Perkin Elmer, Waltham, MA, USA).

### Identification of target genomic locus and validation for effective factors

A locus appropriate for insertion of the pREMI-ZA plasmid on the *K. phaffii* genome was identified as described previously^19^. Genomic DNA from each of the selected strains was extracted using a genome DNA extraction kit (Dr. Gentle, Takara Bio), digested with a mixture of eight restriction enzymes (*Afl*II, *Eco*RI, *Eco*RV, *Hpa*I, *Mlu*I, *Nhe*I, *Spe*I and *Xho*I), blunt-ended using the Klenow fragment (Takara Bio), self-ligated using T4 DNA ligase (Takara Bio) and transformed into the *E. coli* DH5α strain. Transformants with the REMI plasmid pREMI-ZA and a DNA fragment from the *K. phaffii* genome were selected on LB agar plates supplemented with ampicillin (100 μg/mL), followed by replication on LB agar plates supplemented with Zeocin (100 μg/mL). After each re-circularized plasmid was extracted from *E. coli* resistant to both ampicillin and Zeocin, the DNA fragments, including the *K. phaffii* genomic DNA locus flanked by the pREMI-ZA plasmid, were sequenced using an appropriate primer. Based on the genome sequence and annotation in GenBank database for *K. phaffii*^49^, the genomic locus corresponding to plasmid integration of each screening-positive strain was identified.

Targeted gene disruptions of CBS7435_scFv, *dnl4his4*_Pgapdh-scFv and *dnl4his4*_BGL1 strains were performed using a standard homologous recombination method. In brief, three DNA fragments, an antibiotic-resistant resistance cassette (hygromycin, nourseothricin, Zeocin or blasticidin resistance genes, as selective markers), and up- and downstream regions of each of the identified genes (ranging in size from 0.3 to 1.2 kb), were obtained by PCR. DNA fragments for gene disruption (i.e. cassettes consisting of the upstream region, the antibiotic-resistant cassette and the downstream region for each target gene) were prepared by assembly PCR, purified using the Wizard SV Gel and PCR Clean-Up System (Promega), and introduced by electroporation into *dnl4his4* strains previously engineered to secrete recombinant proteins (*i.e*. anti-lysozyme scFv antibody or BGL1p). Transformants with correctly targeted gene disruptions were selected using the appropriate antibiotics and confirmed by colony PCR.

### Cultivation using test tubes or shake flasks

To quantify protein secretion, we first cultivated the yeast strains in test tubes or shake flasks as follows. At least three biological replicate colonies of each yeast strain were streaked on YPD plates supplemented with appropriate antibiotics, then inoculated into 2 mL of BMGY media in test tubes and grown at 30 °C for 24 h at 170 rpm, as a pre-culture step. Pre-cultures in the amount of 200 μL (i.e. 10% seeding) were transferred into 2 mL of BMMY (for *AOX1* promoter strains) or BMGY (for *GAPDH* promoter strains) in test tubes, then cultured at 30 °C for 48 h (for evaluation of the ALE strains) or 60 h (for all other strains, including the screening positive strains and multi-disruption strains) at 170 rpm to induce protein secretion. After cultivation, scFv titer (or BGL1p activity) and an optical density at 660 nm (OD_660_) were measured. For shake-flask cultivation, the (multiple) gene-disruption(s) and host strains were pre-cultured with BMGY in test tubes as described above. Pre-cultures in the amount of 200 μL were inoculated into 20 mL BMMY media in 200 mL buffled shake flasks at an initial OD_660_ = 0.5 and then grown at 30 °C for 72 h. Small aliquots of each culture were sampled at 24 h intervals for ELISA and OD_660_ measurement.

To evaluate protein secretion in each reconstructed yeast strain, we used scFv (or BGL1p) productivity, defined as the scFv titer (or BGL1p activity) per OD_660._ This value provides an indication of the protein secretion ability of specific amount of the yeast cells.

### Next-generation sequencing

Genomic DNA from the screening positive 79G10 strain was prepared from a 4 mL YPD overnight culture solution using a genomic DNA extraction kit (NucleoBond AXG 20 column kit, Macherey-Nagel GmbH & Co. KG, Düren, Germany). The genomic DNA was quantified using a DNA quantification kit (Qubit dsDNA BR Assay kit, Thermo Fisher Scientific). Next-generation sequencing was performed using the Illumina PCR-free library preparation and sequencing protocol according to manufacturer’s instruction. Briefly, genomic DNA (2 ug) was sonicated to 550 bp using an acoustic shearing device (M220, Covaris, Woburn, MA, USA). Fragments were end-repaired, A-tailed and then ligated with the Illumina adapter using a library preparation kit (Truseq PCR free LT Library Prep Kit, Illumina, San Diego, CA, USA). The library was quantified by qPCR using the KAPA Library Quantification Kit for Illumina Libraries (KAPA Biosystems, Wilmington, MA, USA) and library profiles were assessed using a DNA High Sensitivity LabChip kit on an Agilent Bioanalyzer (Agilent Technologies, Santa Clara, CA, USA). The library was sequenced on an Illumina MiSeq instrument using paired-end 300 bp reads.

The MiSeq reads were mapped to the genome of *K. phaffii* CBS7435 (GenBank Accession: FR839628.1) as well as two plasmid sequences (pREMI-ZA and pPAP_scFv) using the Last program^50^ with the options “lastal -Q sanger -f TAB -P 10”. We used an in-house Perl program to extract mutation positions. First, for each read, we obtained the alignment with the best score. If there was more than one alignment with the same best score, a single alignment was randomly selected and used for further analyses. Then, among the genomic positions contained in at least 10 of the alignments, we extracted those positions in which a nucleotide change compared to the reference sequences (i.e. *K. phaffii* genome and plasmid sequences) was observed more frequently than the original nucleotide. Positions in which nucleotide change were observed in at least 90% of the alignments were categorized as *plausible SNP*s and the remaining positions were categorized as *potential SNP*s. We identified SNPs in the 79G10 strain and the host strain separately using the MiSeq reads derived from the respective strain and extracted the positions labeled as plausible SNPs in the 79G10 strain and not labeled as potential SNPs or plausible SNPs in the host strain. Only one SNP passed the criteria; this SNP corresponded to the V50A mutation in the MFα secretion signal in the scFv antibody. The Perl scripts used for the detection of SNPs are available from https://github.com/gterai/snv_detection_PP.

### Determination of mRNA transcript levels by RT-qPCR

The transcript levels for each yeast strain were measured as described in our previous paper^37^. In brief, total RNA was isolated from a 6 h culture after methanol induction (BMMY) using a RNeasy Mini kit (Qiagen, Hilden, Germany). The cDNA templates were synthesized from 0.5 μg of each total RNA using a cDNA synthesis kit (ReverTra Ace qPCR RT master Mix with gDNA Remover, Toyobo, Osaka, Japan). To quantify the target cDNAs, real-time PCR was performed using a SYBR-based qPCR kit (KOD SYBR qPCR Mix, Toyobo) and Mx3005P (Agilent Technologies, Santa Clara, CA, USA). ACT1 was used as an internal standard.

### Western blot analysis

The amounts of scFv antibody secreted by each yeast strain were evaluated by western blot analysis. Equal volumes of supernatants from BMMY cultures at the final culture point (48 h) for each yeast strain were separated by SDS-PAGE. Proteins were transferred to a nitrocellulose membrane using a blotting system (iBlot Gel Transfer System, Thermo Fisher Scientific) according to the manufacturer’s instruction, and non-specific binding was blocked by incubation of the membrane in a blocking reagent (BlockingOne, Nacalai Tesque) at room temperature for 1 h. The blocked membrane was then incubated with rabbit anti-6-His tag antibody (Bethyl Laboratories, Inc., Montgomery, TX, USA) in a TBS-T buffer (10 mM Tris-HCl, pH 8.0, 150 mM NaCl and 0.05% Tween20) at a dilution of 1:5000, at room temperature for 1 h. After washing the membrane with TBS-T buffer, anti-rabbit IgG conjugated with an alkaline phosphatase (Promega) was used for scFv detection, at a dilution of 1:5000. After washing with TBS-T buffer, the antibody-antigen complex was visualized on a CCD imager (LAS-4000, GE Healthcare, Little Chalfont, UK) following addition of a luminescent substrate (CDP-STAR Detection Reagent, GE Healthcare).

### BGL1p activity assay

BGL1p-producing transformants were cultured in test tubes as described in the previous section (Cultivtion using test tubes or shake flasks). The culture media were centrifuged to obtain yeast culture supernatant, which was assayed for BGL1p activity using p-nitrophenyl-β-D-glucopyranoside (pNPG) as the substrate, as reported previously^47^. Briefly, 2 μL of the culture supernatant was added to 98 μL of pNPG solution that had been dissolved in 50 mM sodium citrate buffer (pH 5.0; final pNPG concentration: 2 mM). The solution was mixed and then incubated at 25 °C for 10 min, and then the reaction was stopped by adding 100 μL of 3 M sodium carbonate solution. The amount of p-nitrophenol generated was estimated by measuring the absorbance at 405 nm using an Envision microplate reader (Perkin Elmer).

### Adaptive laboratory evolution (ALE)

Adaptive evolution was performed by serial transfer of test tube cultivated samples. Each of 4 (multiple) gene-disruption(s) and host strains were inoculated into 2 mL of YPG media supplemented with the appropriate antibiotics in test tubes, then grown at 30 °C for 1 day (the host strain, and the single and double gene-disruption strains) or 1.5 days (the triple and quadruple gene-disruption strains). Next 4 μL of each culture was transferred into 2 mL of fresh YPG media supplemented with the appropriate antibiotics. The serial transfer of cultures was repeated 55 times. Samples from these populations were collected periodically and stored in glycerol at −80 °C.

### Growth rate analysis

Growth rates of the gene-disrupted strains were monitored by tracking of OD_660_ values during the exponential growth phase as described in our previous study^33^ except that for this study we used BMGY, YPG supplemented with proper antibiotic(s) (YPG + ) or BMMY media. Briefly, the (multiple) gene-disruption(s) and host strains were streaked on YPD plates supplemented with the appropriate antibiotic(s) and incubated at 30 °C for 2 days. The individual clones were then used to inoculate test tubes containing BMGY or YPG + media. After overnight culture at 30 °C, each culture was diluted to an initial OD_660_ of 0.02 (for inoculation into BMGY and YPG + ) or 0.05 (for inoculation into BMMY) in L-shape tubes containing of the appropriate liquid media (BMGY, YPG + or BMMY). The diluted cells were then cultured at 30 °C using a Bio-photorecorder (TVS062CA; Advantech, Tokyo, Japan) and the OD_660_ was measured at 30 min intervals. Three biological replicates were performed.

### Statistical tests and reproducibility

Two-sided T-tests assuming unequal variance were used to determine the significance of the difference between two groups. *p* values less than 0.05 were considered to be statistically significant. The number of biological replicates or sample size used in this study are more than three and also given in figure legend in detail.

### Reporting summary

Further information on research design is available in the Nature Research Reporting Summary linked to this article.

## Supplementary information

Supplementary Information

Supplementary Data1-5

Supplementary Data6

Reporting Summary

## Data Availability

All data supporting the findings of this study are available within the published article and its supplementary information files. The source data are provided as Supplementary Data 6. The uncropped data of Supplementary Figs. 2c and 5b are shown in Supplementary Fig. 8. The NGS data are deposited in the DDBJ/EMBL/Genbank nucleotide sequence database under accession number DRA013572. The plasmids used in this study can be accessed in Addgene under accession codes 183732, 183733, 183734, 183735 and 183736. All other relevant data are available from the authors upon reasonable request.

## Code availability

Analytical codes for detection of SNPs are publicly available at Github (https://github.com/gterai/snv_detection_PP).
