# Supplementary material for: A streamlined strain engineering workflow with genome-wide screening detects enhanced protein secretion in Komagataella phaffii
Source: Commun Biol. 2022 Jun 8;5:561. doi: 10.1038/s42003-022-03475-w (PMC9177720; doi:10.1038/s42003-022-03475-w)
Supplement: Supplementary file 1 — Supplementary Information [file 42003_2022_3475_MOESM1_ESM.pdf]

Supplementary Information

A streamlined strain engineering workflow with genome-wide screening detects enhanced protein secretion in *Komagataella phaffii*

Yoichiro Ito[1,2], Misa Ishigami[3], Goro Terai[4], Yasuyuki Nakamura[1,2], Noriko Hashiba[3], Teruyuki Nishi[2,5], Hikaru Nakazawa[6], Tomohisa Hasunuma[1,2], Kiyoshi Asai[4], Mitsuo Umetsu[6], Jun Ishii[1,2*] and Akihiko Kondo[1,2,7*]

[1] Engineering Biology Research Center, Kobe University, Kobe, Japan

[2] Graduate School of Science, Technology and Innovation, Kobe University, Kobe, Japan

[3] Technology Research Association of Highly Efficient Gene Design (TRAHED), Kobe, Japan

[4] Department of Computational Biology and Medical Sciences, Graduate School of Frontier Sciences, The University of Tokyo, Chiba, Japan

[5] Biotechnology Research Laboratories, Kaneka Corporation, Takasago, Japan

[6] Department of Biomolecular Engineering, Graduate School of Engineering, Tohoku University, Sendai, Japan

[7] Department of Chemical Science and Engineering, Graduate School of Engineering, Kobe University, Kobe, Japan

[*]To whom correspondence may be addressed.
E-mail: junjun@port.kobe-u.ac.jp
E-mail: akondo@kobe-u.ac.jp

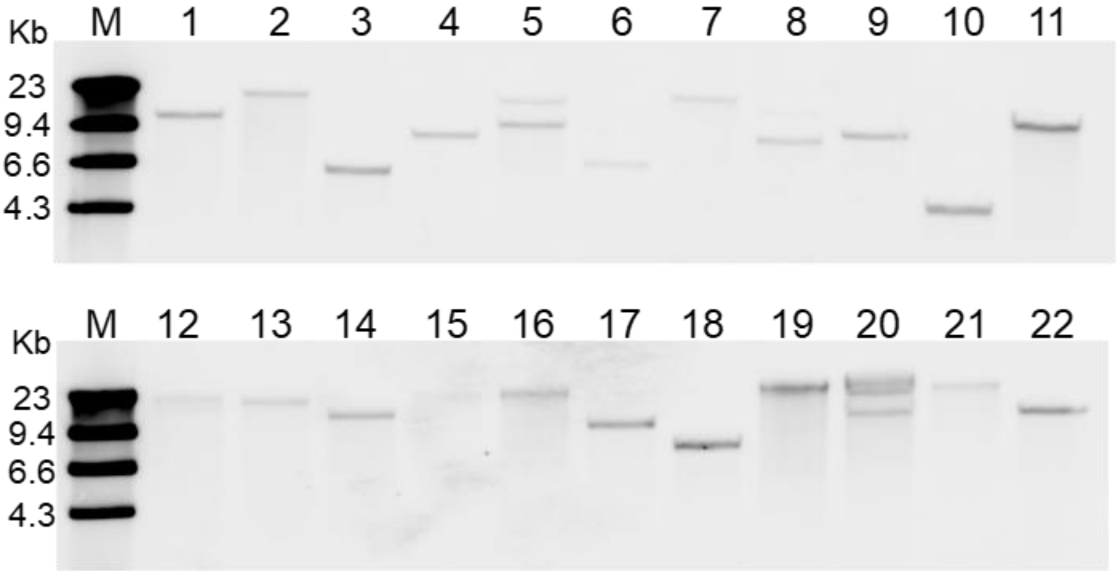

Supplementary Figure 1. Southern blot analysis of randomly picked REMI-based genome-disruption clones. Plasmid DNA (pREMI-ZA) linearized with either *Bam*HI or *Kpn*I was electroporated into the wild-type *K. phaffii* strain CBS7435. Lanes 1-8, *Bam*HI-linearized pREMI-ZA (50 ng DNA); Lanes 9-16, *Kpn*I-linearized pREMI-ZA (50 ng DNA); and Lanes 17-22, *Bam*HI-linearized pREMI-ZA (500 ng DNA). Among the 22 randomly chosen strains, most showed single bands of various mobilities, suggesting that these strains have single, random, independent integrations of pREMI-ZA into the *K. phaffii* genome. The other two strains showed two or more bands, suggesting that these strains harbor two or more integrations. REMI libraries generated using 50 ng DNA in the *Bam*HI and *Kpn*I linearization reactions were used for the screen. These blots indicate uncropped data.

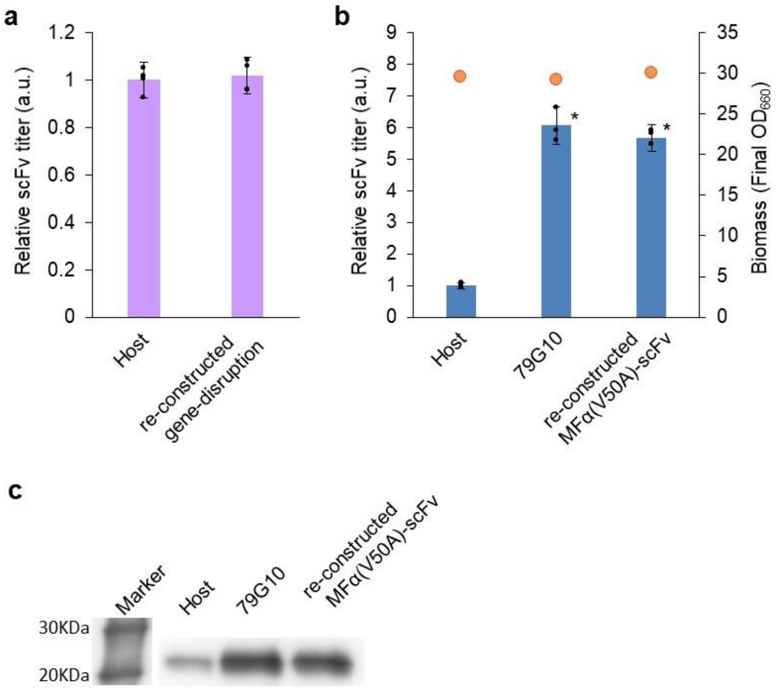

Supplementary Figure 2. A MFα mutation, V50A, results in higher levels of protein secretion. The highest level of secretion of scFv was observed in strain 79G10, which has a REMI plasmid insertion at a gene at chr4-0499. A chr4-0499 gene-disruption strain was newly constructed. The scFv titer in the chr-0499-disruption strain was the same as that of the host strain with non-significance level ($p > 0.05$ by *t*-test) (**a**), indicating that the gene-disruption is unrelated to the change in secretion. In a detailed analysis of a whole genome sequence of the 79G10 strain, we observed a novel V50A mutation on MFα secretion signal. A yeast strain carrying the scFv secretion construct with the V50A mutation on the MFα signal was newly reconstructed. The scFv titer for the reconstructed strain was higher as compared with the host strain and the same as that observed for strain 79G10 (**b**). Western blot analysis of the three strains supports the idea that higher secretion is an effect of the V50A mutation in the MFα signal (**c**). Uncropped data is shown in Supplementary Fig.8. Error bars represent standard deviation from three biological replicates. Asterisks indicate the significance level ($p < 0.05$ by *t*-test) between the host and 79G10 or re-constructed MFα (V50A)-scFv strains.

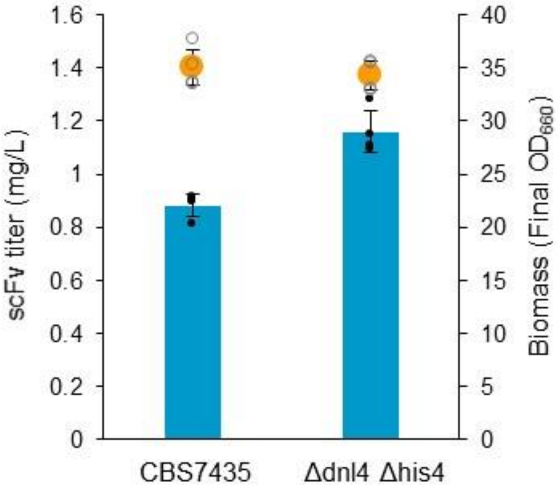

Supplementary Figure 3. The scFv productivities of the *dnl4*-disruption and the CBS7435 host strains. The scFv secretion titers (bars) and final OD values (circles) of the *dnl4his4* and CBS7435 host strain are indicated. Error bars represent standard deviation from four biological replicates. The scFv titer of the *dnl4his4* host strain was significantly higher than that of the CBS7435 derived host strain ($p < 0.05$).

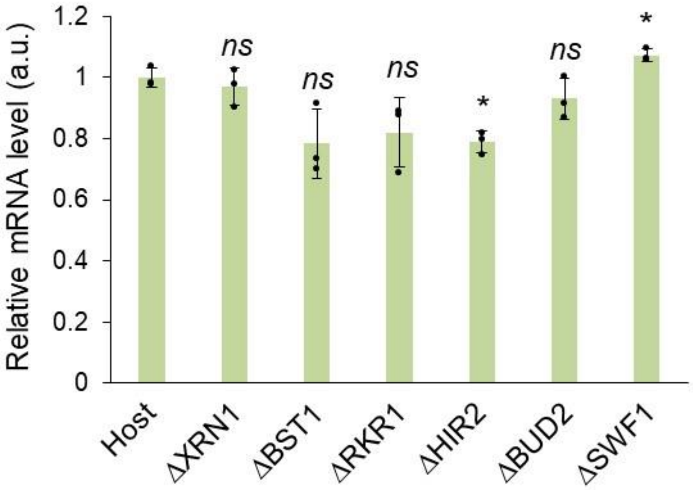

Supplementary Figure 4. Relative mRNA levels. Relative mRNA levels of each reconstructed gene-deletion strain and the host strains were measured by RT-qPCR method. Error bars represent standard deviation from three biological replicates. Asterisks and "*ns*" indicate the significance level ($p < 0.05$ by t-test) and non-significance level ($p > 0.05$ by t-test) between the host strain and the effective factor-disruption strains, respectively.

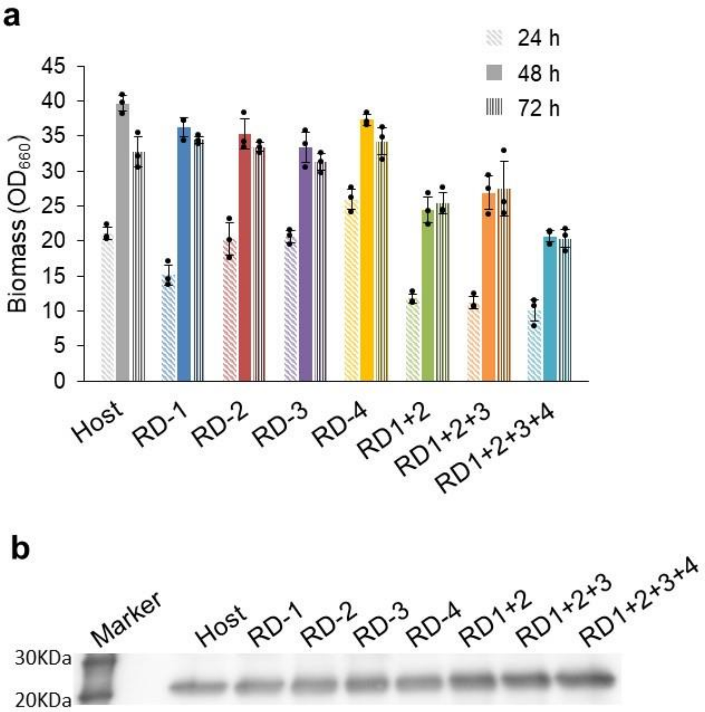

Supplementary Figure 5. Effects of combining multiple gene-disruption of effective factors. (**a**) Biomass (OD_{660}) at 24 h (diagonal stripes), 48 h (filled bars) and 72 h sampling (vertical stripes) after methanol induction in shake flask cultivation are indicated. Gray, blue, red, purple, yellow, yellow-green, orange and blue-green indicate the host, RD-1, RD-2, RD-3, RD-4, RD1+2, RD1+2+3 and RD1+2+3+4 strains, respectively. Error bars represent standard deviations from three biological replicates. The biomass values were raw data used for calculation of scFv productivity shown in Fig. 3. (**b**) Western blot analysis of scFv at 48 h after methanol induction in shake flask cultivation is shown.

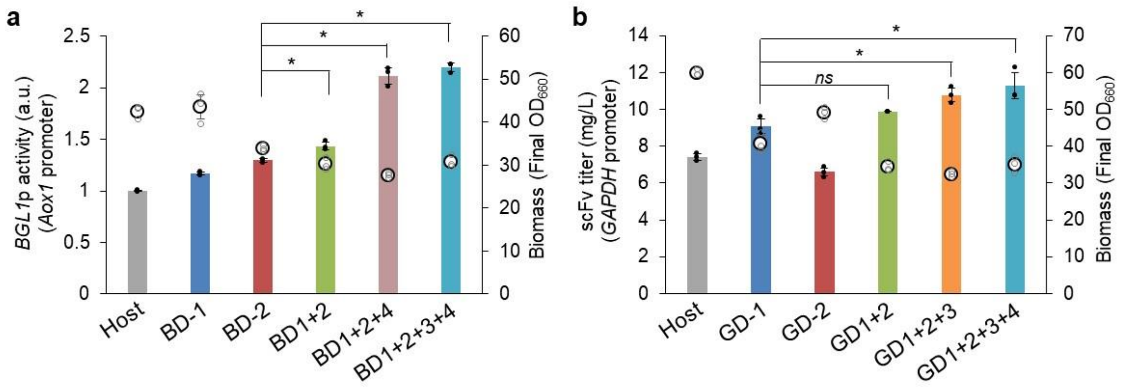

Supplementary Figure 6. Effects of secreted protein-coding gene and promoter swapping in multi-deletion effective factor strains. (**a**) *AOX1* promoter-driven BGL1 construct. (**b**) *GAPDH* constitutive promoter-driven scFv construct. Relative BGL1p activities (**a**, bars), scFv titers (**b**, bars) and final OD_{660} values (open circles, **a** and **b**) of the host, single and multiple gene-disruption(s) strains are indicated. Error bars represent standard deviation from three biological replicates. Asterisks and "*ns*" indicate that a significant level ($p < 0.05$ by *t*-test) and non-significance level between the BD-2 or GD-1 strain (the best secretion strains among single effective factor-disruptions) and the multi-gene-disrupted strains, respectively.

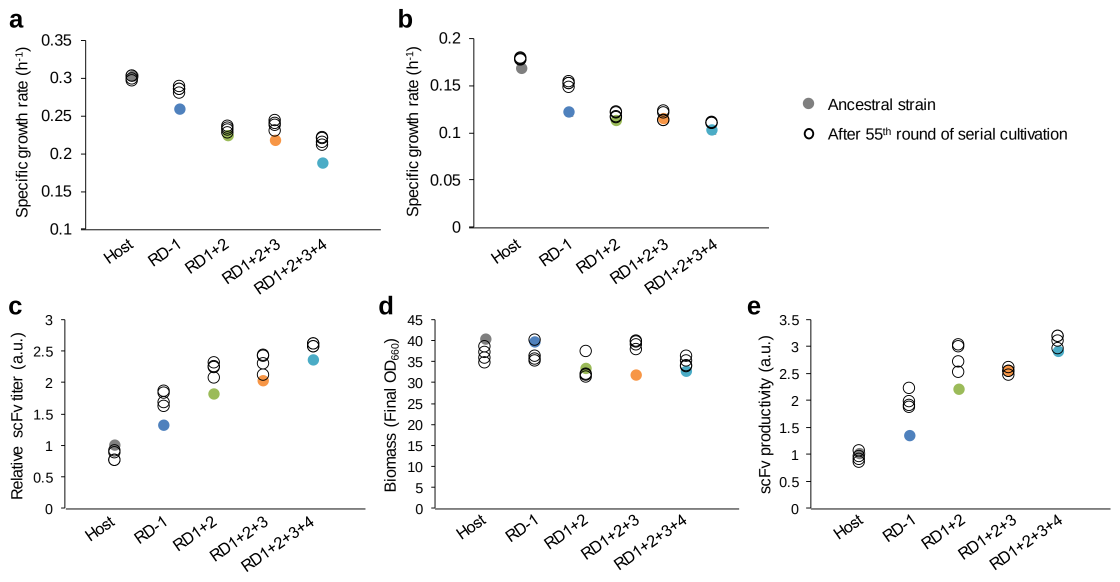

Supplementary Figure 7. Properties of the ancestral and evolved cells in the host and gene-disruption-type effective factor(s) strains. (**a**,**b**) Specific growth rates of the ancestral and evolved strains (four isolated colonies) were evaluated in YPG media supplemented with antibiotic(s) (YPG+) media (**a**) and BMMY media after pre-cultivation in YPG+ (**b**). Adaptive laboratory evolution (ALE) was conducted by performing 55 rounds of serial cultivation in glycerol (YPG+) media. Each of (multiple) gene-disruption(s) of effective factor(s) strain and host strains were inoculated into YPG+. After cultivation at 30°C for 24 h, small aliquots of culture were inoculated into YPG+ (initial OD_{660}: 0.02) (**a**) and BMMY (initial OD_{660}: 0.05) (**b**) followed by cultivation at 30°C with a Bio-photorecorder. Relative scFv titer (**c**), biomass (final OD_{660}) (**d**) and relative productivity (**e**) of the ancestral and evolved strains (four isolated colonies) grown in BMMY media for 48 h are shown. Filled and open circles indicate the ancestral strain and four isolated strains, respectively, after the 55th-serial cultivation. Colors were used as in Fig.4 (Gray, blue, yellow-green, orange and blue-green indicate the host, RD-1, RD1+2, RD1+2+3 and RD1+2+3+4 strains, respectively). Each titer and productivity value was normalized to that of the ancestral host strain. Error bars represent standard deviation from three biological replicates.

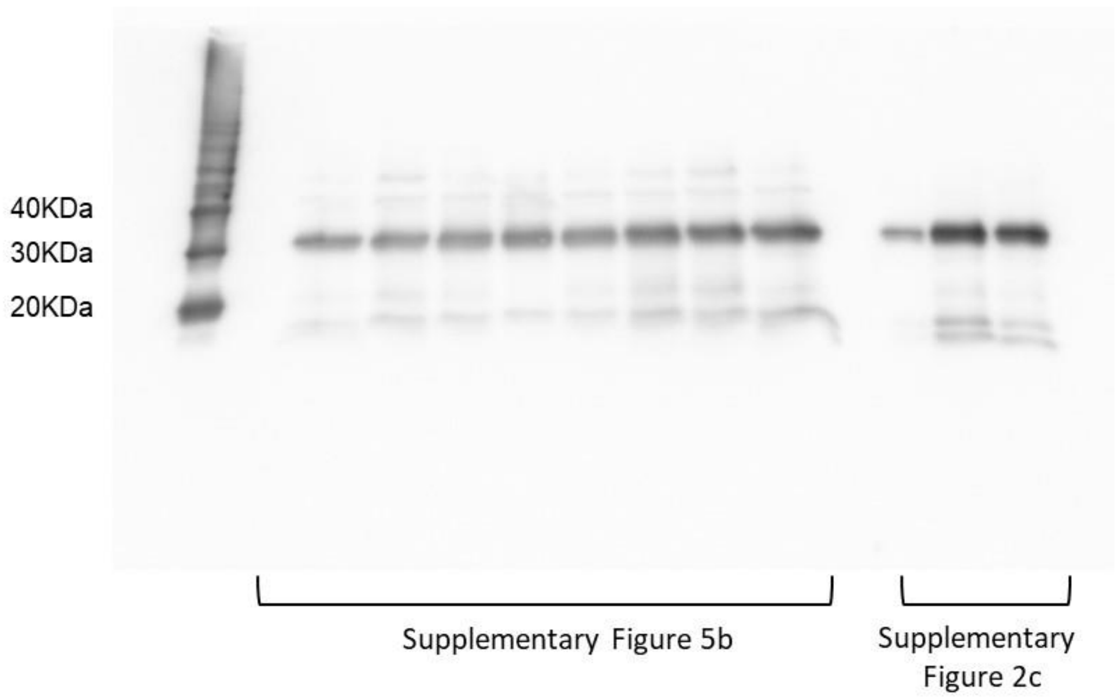

Supplementary Figure 8. Uncropped and unedited images for Supplementary Figure 2c and 5b.
